# Universal Revaccination

**Published:** 1902-08-16

**Authors:** 


### UNIVERSAL REVACCINATION.

Speaking at the Manchester meeting of the British Medical Association, Dr. Edward J. Edwardes pointed out the absurdity of the English method of dealing with small-pox. After showing how Germany had been able, by adopting compulsory revaccination, to wipe out small-pox, and how the experience both of England and of other countries had sufficed to show that once vaccination was utterly insufficient for the control of the disease, he said that while the Germans had had the intelligence to grasp this and to act upon it by adopting obligatory revaccination for all children, England had apparently not recognised the bearing of the facts, but went blundering on, spending thousands here and thousands there on isolation hospitals which ought to be unnecessary. A large small-pox hospital in the present state of knowledge was, he said, an anachronism and an absurdity. "We wait till people get infected and then remove them; we are running after the disease instead of preventing it, as we can by revaccination." Dr. Edwardes also insisted upon the fallacy of the argument maintained by some that those who believe in revaccination should get revaccinated, but should leave the rest alone, and pointed out how definitely the statistics of the German army went to show that revaccination must be universal if it is to abolish small-pox epidemics. The abolition of small-pox in the German army was not entirely due to the enforcement of revaccination in its own ranks, but to the fact that after 1874 the army found itself amongst a well-protected population. "Thus, it is of direct importance to the whole community," he said, "that each individual should be well protected, and since everything must give way to the good of the whole community, this gives the right to the State, as representing the community at large, to insist on universal revaccination."

